# Apple and Sugar Feeding in Adult Codling Moths, *Cydia pomonella*: Effects on Longevity, Fecundity, and Egg Fertility

**DOI:** 10.1673/031.011.16101

**Published:** 2011-11-22

**Authors:** Erik J. Wenninger, Peter J. Landolt

**Affiliations:** ^1^ Department of Plant, Soil, and Entomological Sciences, University of Idaho, Kimberly Research and Extension Center, Kimberly, ID 83341-5082, USA; ^2^ Department of Plant, Soil, and Entomological Sciences, University of Idaho, Twin Falls Research and Extension Center, Twin Falls, ID 83303-1827, USA

**Keywords:** diet, feeding behavior, lifespan, oviposition, reproduction

## Abstract

Attraction of adult codling moths, *Cydia pomonella* (L.) (Lepidoptera: Tortricidae), to sweet baits has been well documented. However, beneficial effects of sugar feeding on moth fitness have not been demonstrated. Longevity, fecundity, and egg fertility were examined for female/male pairs of moths maintained with the following food regimens: water, sucrose water, honey water, apple juice, apple flesh, or starved, i.e., no food or water provided. Longevity and total fecundity were enhanced in all treatments relative to the starved treatment moths. Sucrose water, honey water, and apple juice treatments yielded the highest longevity, but total fecundity was highest for moths maintained on honey water or apple juice. Total egg fertility did not differ among treatments. However, egg fertility declined more gradually over the female lifespan for the three aqueous solution diets of sucrose water, honey water, and apple juice. Similarly, fecundity per day declined more gradually over time for honey water and apple juice treatments. Performance of moths maintained with apple flesh was generally intermediate between that of moths with water and the three aqueous solution treatments. This suggests that moths benefit from feeding on ripe apple flesh, although apple may be more difficult to ingest or its nutrients less concentrated compared to aqueous solutions. The results presented here may explain attraction of adult moths to sweet baits as well as to odors from ripe fruit, which may be a natural source of food in the fall.

## Introduction

In the state of Washington, codling moths, *Cydia pomonella* (L.) (Lepidoptera: Tortricidae), typically exhibit two generations per year, with the first adult flight during May to June and the second from late July to early September (Knight and Light 2005). Because apples and pears do not typically begin to ripen until August, most adult activity, including oviposition by females, occurs when trees have immature fruit. This is not surprising given that *C. pomonella* larvae do not exhibit normal development on the flesh or seeds of ripe apples (Heriot and Waddell 1942). Despite the usual development of *C. pomonella* larvae on immature fruit, Landolt and Guédot (2008) reported that adults of both sexes are attracted to traps baited with ripe apples or pears. Moreover, ethyl (*E,Z*)-2,4-decadienoate (pear ester)—a key odor of ripe pear fruit (Light et al. 2001)—has been shown to be a potent attractant to both male and female *C. pomonella* (Light et al. 2001; Thwaite et al. 2004; Trimble and El-Sayed 2005), especially when combined with acetic acid, a fermentation byproduct (Landolt et al. 2007). In light of larval dietary requirements and the phenology of codling moth oviposition, the attraction of adults to odors from ripe fruit is an anomaly that remains to be explained. Landolt and Guédot (2008) hypothesized that codling moth response to ripe fruit odors might be explained as the seeking of feeding sites.

Many species of moths feed on sugar sources such as floral and extrafloral plant nectars, saps, honeydews, fruit, and fruit juices (Norris 1936). However, information on adult feeding in *C. pomonella* is limited, and there are no known published accounts of adults feeding on ripe fruit in nature. Attraction of adult *C. pomonella* to syrups, fruit juices, sweet baits, and acetic acid—a microbial fermentation product in sweet baits (Utrio and Eriksson 1977)—has been well documented (Yothers 1927; Eyer 1931; Dethier 1947), but the biological importance of this attraction remains unclear.

Geier (1963) stated that *C. pomonella* readily feed on sugar solutions, but investigations into effects of adult feeding on moth biology have yielded inconsistent results. Wiesmann (1935) reported that females fed honey water laid twice as many eggs as starved moths, and that post-emergence oogenesis was dependent on water consumption in the adult stage. However, Howell (1981) reported that neither fecundity nor egg fertility was enhanced significantly by access to water or aqueous sugar solutions, but feeding did increase longevity. Because the number of fertile eggs laid by *C. pomonella* decreases with increased female age (Deseö 1971; Vickers 1997), any enhancement of longevity from feeding may be expected to result in minimal increase in reproductive output. According to Benz (1991), most adult Tortricidae do not require sugars to achieve normal longevity and fecundity. For example, *Epiphyas postvittana* exhibited greater longevity and fecundity when provided water, but showed no additional benefit when provided honey water (Gu and Danthanarayana 1990), and *Cydia molesta* showed no increase in longevity or fecundity when provided with sugar (Benz 1991). However, several tortricid species have been shown to exibit higher fecundity and in both sexes greater longevity when given access to sucrose and/or honey water (Savopoulou-Soultani et al. 1998; Stevens et al. 2002), or extrafloral nectar (Atanassov and Shearer 2005). Moreover, the tortricid *Homona coffearia* failed to produce any eggs without access to sugar (Sivapalan et al. 1977).

This study investigates how adult feeding in *C. pomonella* affects longevity, fecundity, and egg fertility by comparing these parameters among adult moths maintained with different diets. The results presented here may explain attraction of adult moths to sweet baits as well as to ripe fruit, which may be a source of food in nature.

## Materials and Methods

Codling moths were held in 473 ml plastic cups (Solo Cup Company, www.solocup.com) with lids modified with a fine mesh screen to provide ventilation. We placed a 70 mm diameter piece of filter paper on the bottom of each cup to absorb possible spilling of feeding solutions, and we affixed a yellow sticky note on the inside wall of each cup to provide a rough surface on which moths could walk in case the interior of the plastic cups became covered in scales. We obtained adult moths within 24 hours of eclosion from the Yakima Agricultural Research Laboratory, Wapato, WA colony and established female/male pairs of moths in the plastic cups. Moths were held under a 16:8 L:D photoperiod at 24° C and 65% relative humidity.

Each pair of moths was provided with one of the following treatments: no food or water (starved); water alone; aqueous 13% sucrose solution; aqueous 15.4% raw honey solution; apple juice; slice of ripe apple flesh, ∼ 6 g. For each treatment 24–29 pairs of moths were observed. Deionized water was used for the water only treatment and for the aqueous solutions. Water and aqueous solutions were provided on a saturated cotton ball placed in a plastic dish; identical dishes without cotton balls were also provided in the starved and apple slice treatments. The apple slices were Braeburn variety apples from the previous Washington crop, purchased from a local grocery store and kept in cold storage until used in experiments. The apple juice was pasteurized, fresh pressed juice from Granny Smith, Red Delicious, and Golden Delicious varieties of apples with no additives (Tree Top, www.treetop.com). Concentrations for the sucrose and honey solutions were chosen to yield ∼ 13% total sugar content, which is similar to the ∼ 13.5% total sugar content found in ripe Braeburn variety apples (Hecke et al. 2006); honey contains ∼ 82% total sugars (USDA-ARS 2009). The apple juice contained ∼ 11% sugar. Food and water were replaced every two days.

All moths were transferred to new cups 6–7 days after initiation of treatments and every six days thereafter. After moths were transferred to new cups, all eggs within each cup were counted and held for up to 10 days to assess egg fertility. Neonate larvae were counted and removed from cups every 1–2 days. Adult moths were observed daily to determine longevity.

Data were transformed as necessary to achieve normality and equal variance. Analysis of variance (ANOVA) was used to compare adult longevity (log transformed) among feeding treatments and between the sexes. Additionally, ANOVA was used to compare total number of eggs laid and percentage of eggs laid that were fertile (Box— Cox transformed, λ = 1.5) among treatments, with female longevity—a trait related to fecundity and egg fertility—included in the model. Where ANOVA showed significant differences, the Ryan, Einot, Gabriel, and Welsch multiple range tests were used to discriminate among treatments (Ryan 1959, 1960; Einot and Gabriel 1975; Welsch 1977).

**Table 1.**
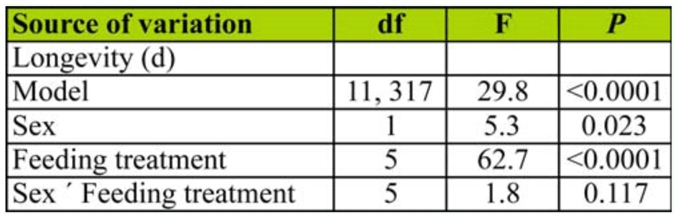
ANOVA examining longevity (log transformed) of *Cydia pomonella* as a function of feeding treatment and sex.

To examine the effects of feeding treatment on the number of eggs laid per female per day over each of the egg collection dates, repeated measures ANOVA were used with a first order autoregressive covariance structure. The model included the interaction between the main effect and the time factor. The effects of feeding treatment over time on egg fertility (arcsin transformed) were analyzed similarly with repeated measures ANOVA. Where repeated measures ANOVA showed significant differences, Fisher's least significant differences tests were conducted to discriminate among treatments. All statistical analyses were performed using SAS (SAS Institute 2002). Statistical significance level was set at a = 0.05.

## Results

**Figure 1.**
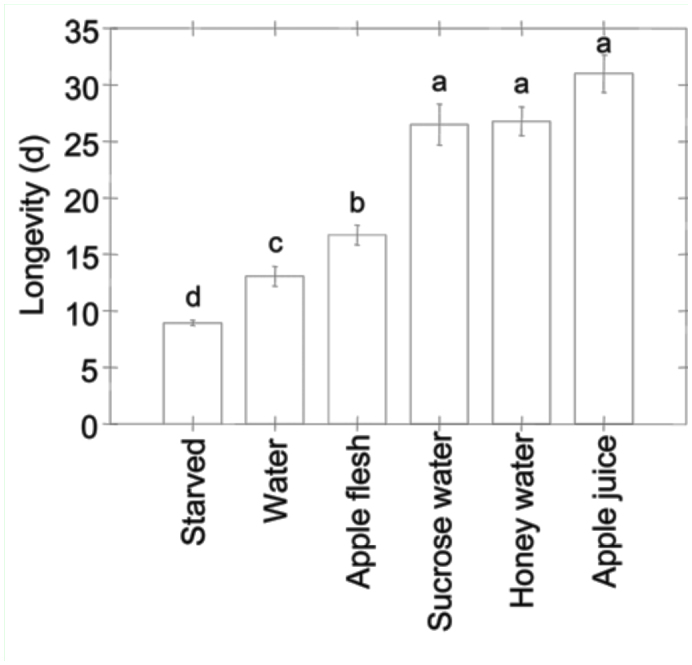
Effect of feeding treatment on mean moth longevity; male and female *Cydia pomonella* were analyzed together. Means that do not share the same letter differ significantly based on REGW multiple range tests (α = 0.05). N = 24–29 pairs of moths per treatment. Error bars represent standard error. High quality figures are available online.

Longevity of adult moths differed significantly among feeding treatments and between the sexes, though the interaction term was not statistically significant (Table 1). Mean ± SEM female longevity (19.1 ± 0.9 days) was shorter than that of males (21.6 ± 1.0 day). Sucrose water, honey water, and apple juice treatments yielded the highest overall longevity. Longevity of moths provided apple flesh was higher than that of moths provided water, which was higher than that of moths that were starved (Figure 1).

**Figure 2.**
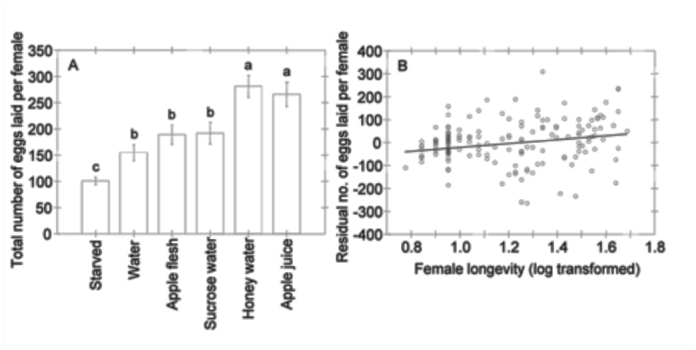
(A) Effect of feeding treatment on mean female *Cydia pomonella* fecundity and (B) effect of female longevity on fecundity, accounting for effects of feeding treatment. Plotted in (B) is the relationship between female longevity and the deviation from expected fecundity due to feeding treatment, i.e., the residuals following the ANOVA of feeding treatment effects on fecundity. Means comparisons and error bars as in Figure 1. High quality figures are available online.

The total number of eggs laid by females differed significantly among feeding treatments and was positively correlated with female longevity (Table 2, Figure 2). Total fecundity was highest for moths maintained on honey water or apple juice (Figure 2A). Total fecundity did not differ among sucrose water, apple flesh, and water treatments. Starved moths laid fewer eggs than in all other treatments, although the difference between the starved and water treatments was not significant (Figure 2A).

**Table 2.**
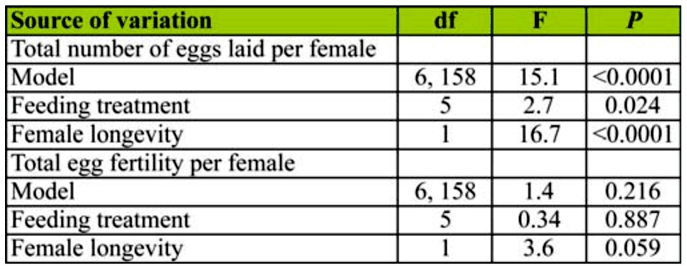
ANOVAs comparing total number of eggs laid by female *Cydia pomonella* or total egg fertility (Box—Cox transformed, λ = 1.5) as a function of feeding treatment and female longevity (log transformed).

**Table 3.**
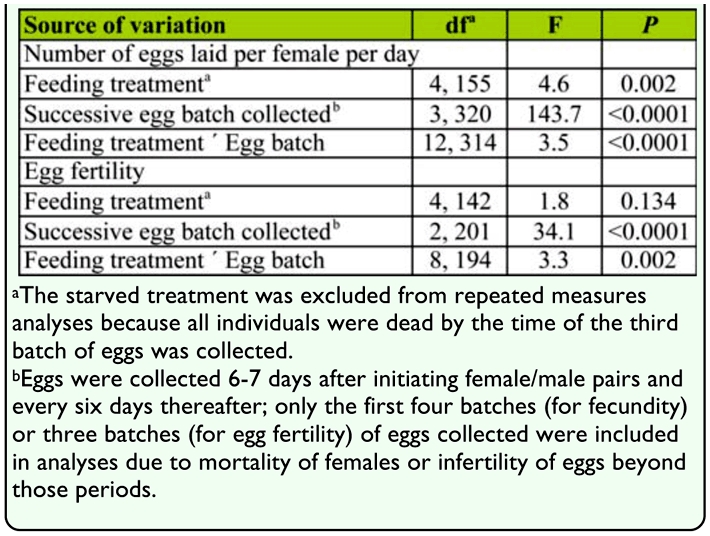
Repeated measures ANOVAs comparing number of eggs that female *Cydia pomonella* laid per day or egg fertility (Box— Cox transformed, λ = 1.5) as a function of feeding treatment over the first four (for number of eggs laid) or three (for egg fertility) successive batches of eggs collected.

**Figure 3.**
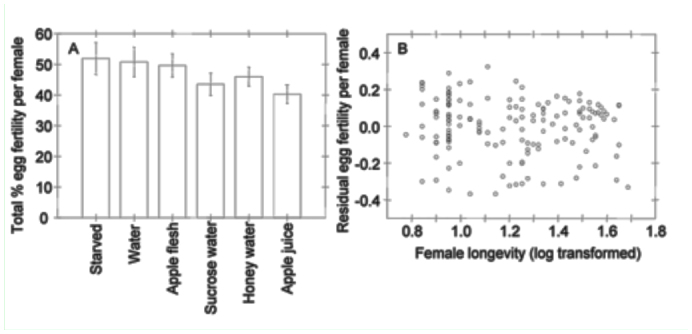
(A) Effect of feeding treatment on mean % egg fertility of *Cydia pomonella* and (B) effect of female longevity on % egg fertility, accounting for effects of feeding treatment. Plotted in (B) is the relationship between female longevity and the deviation from expected % egg fertility due to feeding treatment, i.e., the residuals following the ANOVA of feeding treatment effects on % egg fertility. Means comparisons and error bars as in Figure 1.High quality figures are available online.

Total egg fertility did not differ among feeding treatments, nor did it vary significantly with female longevity. However, total egg fertility tended to decline with increasing female longevity (Table 2, Figure 3).

**Figure 4.**
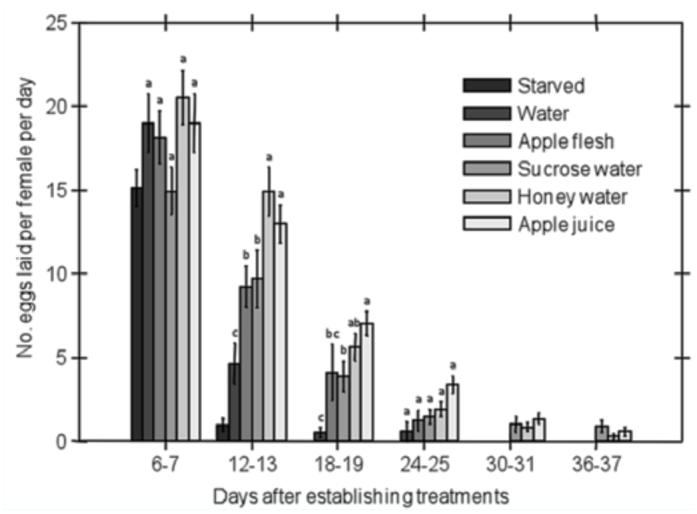
Effect of feeding treatment on female fecundity of *Cydia pomonella* over time. Fecundity was assessed 6–7 days after initiation of treatments and every six days thereafter. Data from the starved treatment and from egg batches beyond 25 days were excluded from the analysis due to heavy mortality. Means within each time period that do not share the same letter differ significantly based on least significant difference tests (α = 0.05). Error bars represent standard error. High quality figures are available online.

The number of eggs laid by females per day significantly differed among feeding treatments and among batches of eggs collected over time. The interaction between feeding treatment and the time factor was also significant (Table 3), displaying a decline in fecundity per day across all feeding treatments over time, though a more gradual decline was shown for both apple and aqueous solution treatments. The honey water and apple juice treatments in particular showed a more gradual decline in fecundity relative to the other treatments (Figure 4). For example, in the second batch of eggs collected 12–13 days after treatments were established, moths maintained on apple juice or honey water laid significantly more eggs than moths maintained on sucrose water or apple flesh, which in turn laid significantly more eggs than moths maintained on water alone (Figure 4).

**Figure 5.**
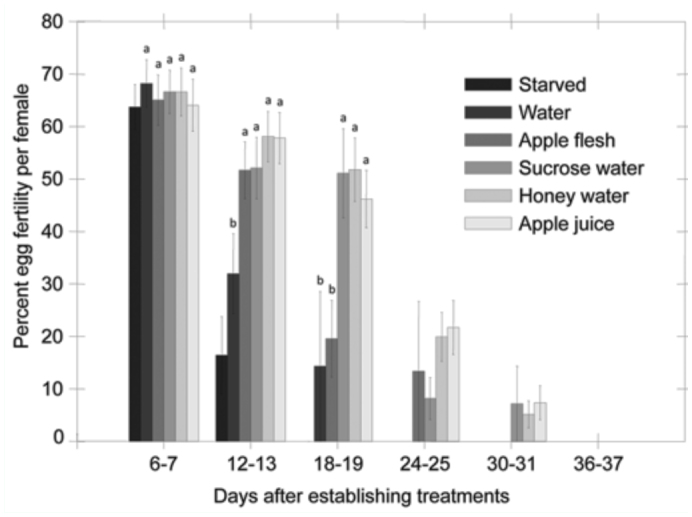
Effect of feeding treatment on % egg fertility of *Cydia pomonella* over time. Egg fertility was assessed 6–7 days after initiation of treatments and every six days thereafter. Data from the starved treatment and from egg batches beyond 19 days were excluded from the analysis due to limited egg fertility. Means comparisons and error bars as in Figure 4. High quality figures are available online.

Egg fertility did not differ among feeding treatments, but did vary over time; the interaction between feeding treatment and the time factor was significant (Table 3). Egg fertility declined across all feeding treatments over time, though more gradually for the apple flesh and sugar solution treatments. The sucrose water, honey water, and apple juice treatments in particular showed a more gradual decline in fecundity relative to the water treatments (Figure 5). For example, for the third batch of eggs collected 18–19 days after treatments were established, moths maintained on apple juice, honey water, or sucrose exhibited greater egg fertility than moths maintained on apple flesh or water (Figure 5).

## Discussion

Attraction of adult *C. pomonella* to sweet baits and to odors from ripe fruit is well documented, but it remains to be determined whether this attraction can be explained by an advantage conferred to moths by feeding. Attraction to ripe fruit in relation to the seeking of oviposition sites is not expected because nearly all oviposition by *C. pomonella* in the field occurs before fruit on trees ripen (Knight and Light 2005), and larvae are unable to develop normally on ripe fruit (Heriot and Waddell 1942). The data presented in the current study support the hypothesis that adult moths of both sexes are attracted to sweet baits and odors from ripe fruit because of the beneficial effects of adult feeding.

Male attraction to these potential food odors also could be explained in part in relation to seeking reproductively mature females, as suggested by Landolt and Guédot (2008). For example, pear ester, an odorant of ripe pear fruit, is more attractive to males than to females (Thwaite et al. 2004), in some studies is exclusively attractive to males (Kutinkova et al. 2005), and is co-attractive with acetic acid in attracting male and female *C. pomonella* (Landolt et al. 2007). Although it is suggested that codling moth response to pear ester may be a means to locate an adult food source, it should also be considered that males might orient to these cues as a means of locating feeding females. Whether mate-finding behavior plays a role in attraction to ripe fruit odors or other fermented sugar sources remains to be explored.

The results reported here provide evidence that adult moths of both sexes benefit from feeding on water, apple flesh, and aqueous sugar solutions. Longevity of moths maintained on ripe apple flesh generally was intermediate between that of moths provided water versus those provided with one of the three sugar solution treatments. These results suggest that moths benefit from feeding on ripe apple flesh, but may be expending more time and energy by feeding on apple flesh and/or are obtaining fewer nutrients from apple flesh compared to apple juice or aqueous solution treatments. Sugar feeding by females in the field might enhance fecundity and egg fertility directly, and increased longevity from feeding also might extend oviposition periods. Wiesmann (1935) reported that starved females had more mature, unlaid ova in their bodies at death than did honey-fed females. This suggests that part of the benefit of water or sugar consumption derives from increased longevity, allowing for a longer oviposition period. In males, enhanced longevity from feeding might lead to more mating opportunities. It is also possible that sugar consumption could enhance moth dispersal. Of the tortricids studied to date, energy for flight in most species is derived from lipid reserves, but carbohydrates may be used in some species (Benz 1991). Moths exhibited higher fecundity and egg fertility in apple juice and honey treatments than in the sucrose treatment, which suggests that other nutrients found in apple juice (vitamins, minerals, amino acids) and honey (galactose, maltose, vitamins, minerals, amino acids) (USDA-ARS 2009) might contribute to adult moth nutrition.

The extent to which fecundity of *C. pomonella* would be enhanced by feeding in nature depends in part on whether the longevities recorded in the laboratory can be extrapolated to those in the field. It is unlikely that many moths in the field would live for the durations that were observed for moths maintained on aqueous diet solutions in the laboratory. However, on a degree-day basis, longevities of moths that were starved or maintained on water were similar to longevities recorded by Jones and Wiman (2008) for unfed *C. pomonella* caged in the field (data not shown). Moths maintained on water or any of the food treatments showed enhanced fecundity and egg fertility relative to starved moths after the first week. Codling moths should be expected to derive some benefit from food or water sources available in the field, even if their longevities are enhanced only moderately.

The clear benefits of feeding to female reproductive output observed in the experiments described here are at odds with a previous study that reported sugar feeding in *C.* *pomonella* to increase longevity but not fecundity or egg fertility (Howell 1981). However, the increase in fecundity from sugar feeding that we observed is consistent with an earlier report of enhanced fecundity with honey feeding (Wiesmann 1935). In the present study, effects of feeding on egg fertility were only evident over time. No overall effects of feeding on egg fertility were evident because fed females lived longer and laid more eggs; these eggs were increasingly less likely to be fertile as the females aged. This pattern obscured the beneficial effects of feeding on egg fertility and underscores the importance of considering subtle temporal effects of feeding in addition to more broad effects.

Of interest from both fundamental and applied perspectives is the relationship between attraction of moths to sweet baits and moth sex, age, and mating status. Yothers (1927) reported that nearly all of females captured with sweet and fermented baits were gravid; presumably captured soon after emergence and before they laid many of their eggs. Landolt and Guédot (2008) reported that a majority of females collected in traps baited with ripe apples or pears were mated and possessed unlaid eggs or ova, and fewer females were post-reproductive or unmated. These trap catch patterns are consistent with the strong relationship between adult feeding and reproductive output observed in the present study. Further clarification of how age and reproductive state relates to attraction of *C.* *pomonella* to pear ester, acetic acid, and other putative feeding attractants will be necessary to understand the value of these attractants as possible monitoring and management tools. For example, if a feeding attractant is attractive to females soon after eclosion and before many eggs are laid, it may be useful in an attract-and-kill application.

Although Dethier (1947) stated that *C. pomonella* feed on nectar and fermenting fruit, no detailed observations or data were presented. Moreover, there are no known published details of adult *C. pomonella* feeding on ripe or over-ripe fruit in nature, nor of any evaluations of adult feeding on fruit in the laboratory. Interestingly, Wiesmann (1935) did observe adult *C.* *pomonella* feeding on water droplets and aphid honeydew in the field. Because sugar feeding is common in many insect groups including the Lepidoptera (Norris 1936; Wackers et al. 2007), and attraction of *C. pomonella* to sweet baits has been well documented (Yothers 1927; Eyer 1931; Dethier 1947), it is not surprising that *C.* *pomonella* feed on sugars. In laboratory experiments with *C. pomonella*, sucrose or honey solutions are often supplied to adults despite limited evidence for beneficial effects on moth biology. The results presented here represent a robust demonstration that *C. pomonella* show enhanced longevity and reproductive output from sugar feeding. These results may explain attraction of adult moths to sweet baits and odors from ripe fruit, which may be a natural source of food in the field.
